# Risdiplam therapy in adults with 5q-SMA: observational study on motor function and treatment satisfaction

**DOI:** 10.1186/s12883-024-03562-x

**Published:** 2024-02-17

**Authors:** Bogdan Bjelica, Camilla Wohnrade, Iraima Cespedes, Alma Osmanovic, Olivia Schreiber-Katz, Susanne Petri

**Affiliations:** 1 Department of Neurology, Hannover Medical School, 1, Carl-Neuberg-Straße, Hannover, 30625 Germany; 2 Essen Center for Rare Diseases (EZSE), University Hospital Essen, Essen, Germany

**Keywords:** Spinal muscular atrophy, Risdiplam, Motor function, Adverse events, Treatment satisfaction

## Abstract

**Background:**

We aimed to describe the experience of a single neuromuscular center in Germany in treating adult spinal muscular atrophy (SMA) patients with risdiplam and to analyze motor function and treatment satisfaction during a follow-up period up to 20 months.

**Methods:**

Fourteen patients with type 2 or 3 SMA (seven with SMA type 2, six with SMA type 3; age range: 18–51) were included. The Revised Upper Limb Module (RULM) and the Hammersmith Functional Motor Scale Expanded (HFMSE) were recorded at baseline and at follow-up (month 4, 8, 12, 16, 20). Treatment adverse events were collected at every follow-up visit. Patients’ treatment satisfaction was assessed by the Treatment Satisfaction Questionnaire for Medication (TSQM).

**Results:**

Half of the patients reached the 20-month follow-up. Based on the HFMSE score, no patients had clinically meaningful improvement. Twelve remained stable (92.3%), two showed transient clinically meaningful deterioration (15.4%) and one experienced lasting clinically meaningful deterioration (7.7%). Based on the RULM scores, seven patients were either stable or demonstrated clinically meaningful improvement (53.8%) and six showed clinically meaningful deterioration (46.2%). There was no treatment withdrawal during the follow-up. The most common adverse events were skin rash/increased skin sensitivity to sunlight (*n* = 3), diarrhea (*n* = 3), aphthous ulcer (*n* = 3) and abdominal pain (*n* = 2). Most patients stated to be at least “satisfied” with the medication.

**Conclusions:**

Risdiplam was well tolerated. Half of the patients remained stable or improved after risdiplam initiation. Larger and multicentric studies are needed to better understand the long-term effects of risdiplam in adult SMA.

## Introduction

5q-spinal muscular atrophy (SMA) is one of the most common genetic diseases with autosomal recessive inheritance. It has an estimated incidence of 1 in 6000 to 1 in 10000 live births. SMA mainly affects lower motor neurons and is therefore characterized by progressive and predominantly proximal muscular weakness and atrophy [1]. Homozygous mutations in the exons 7 and/or 8 of the *survival of motor neuron* (*SMN*) *1* gene located on chromosome 5q13.2 are the cause of SMA [2, 3]. Due to alternative splicing of the paralogous *SMN2* gene pre-mRNA transcript and exclusion of exon 7, low level of functional SMN protein is produced, not sufficient to compensate for the deficit of the SMN protein [1]. Based on the age at symptom onset and motor milestones achieved, SMA can be classified into five groups (SMA type 0 to 4). A milder disease phenotype is associated with higher *SMN2* copy numbers [4].

Currently, there are three approved disease-modifying therapeutic options increasing the production of SMN protein: the intrathecally administered antisense oligonucleotide nusinersen [5, 6] intravenously administered adenovirus-associated gene replacement therapy with onasemnogene abeparvovec-xioi [7, 8] and risdiplam. Risdiplam is the first orally available drug and has been approved by the US Food and Drug Administration in 2020, and the European Medicines Agency in 2021 for patients with SMA type 1, 2, or 3 and/or carrying 1–4 *SMN2* gene copies [9, 10]. It is an SMN2 pre-mRNA splicing modifier which promotes inclusion of exon 7 and thereby increases the production of functional SMN protein [11]. Safety, tolerability, and efficacy was evaluated in two multinational, double-blind, randomized, placebo-controlled, phase 2/3 trials: FIREFISH [12] and SUNFISH [13]. While part 2 of the FIREFISH trial showed that risdiplam given once daily improved motor function and survival in 41 infants with SMA type 1 [12], part 2 of the SUNFISH trial (180 included non-ambulatory SMA type 2 and 3 patients aged 2–25 years) revealed improvement of motor function after 24 months of treatment, compared to untreated patients. The biggest benefits were observed in the youngest patients (2–5 years), while no improvement was seen in the oldest age group (18–25 years) [13]. However, stabilization or improvement was observed across all age groups [13]. In the SUNFISH trial the authors reported no treatment-related adverse effects that led to withdrawal or treatment discontinuation during 24 months of treatment.

To date, there are two previous reports on motor function during risdiplam therapy in adult SMA patients in a real-world setting, each comprising six patients with SMA type 2 [14, 15]. McCluskey et al. [14] showed no change in the Revised Upper Limb Module (RULM) score after 6 months of treatment, while Ñungo Garzón et al. [15] observed improvements in RULM in two out of six non-sitter patients > 16 years of age after 12 months. Larger multicentric and longitudinal data regarding safety, tolerability, and efficacy of risdiplam in adults with SMA are lacking.

The aim of the present study was to describe the experience of a single neuromuscular center in Germany in treating adult SMA patients with risdiplam and to analyze treatment safety, motor function and treatment satisfaction during a follow-up period up to 20 months.

## Materials and methods

### Participants

All patients with SMA who received risdiplam treatment at the Department of Neurology of Hannover Medical School between April 2021 and February 2023 were included in this prospective, longitudinal, monocentric, observational study. All patients had a genetically confirmed diagnosis of SMA and were 18 years or older. Treatment with risdiplam was prescribed according to the recommendations. The SMA cohort consisted of 14 patients regularly visiting the neuromuscular clinic of Hannover Medical School. Sociodemographic and clinical data were collected at baseline, including gender, age at treatment initiation, previous therapy with nusinersen, disease duration, SMA type, *SMN2* gene copy number, ability to walk (defined as at least 10 m without assistance or use of a device such as cane or a walker [16]), presence of scoliosis, use of non-invasive ventilation (NIV) and presence of percutaneous endoscopic gastrostomy (PEG). For patients previously treated with nusinersen, risdiplam was started at least four months after the last dose of nusinersen. Follow-up data were collected four, eight, 12, 16 and 20 months after initiation of risdiplam treatment. To evaluate safety, individual treatment side effects and laboratory assessments were recorded at every follow-up visit. Regarding safety analysis, no follow-up data was lost. Regarding the analysis of motor function: at baseline three patients did not attend the appointment for the analysis of motor function, at month 4 two patients refused to be tested, at month 8 one patient refused to be tested and two patients did not attend the appointment for the analysis of motor function, at month 12 three patients did not attend the appointment for the analysis of motor function and at month 16 one patient did not attend the appointment for the analysis of motor function. Patients provided various reasons for not attending scheduled appointments or refusing testing at specific time points. These reasons included the perceived time-consuming and burdensome nature of motor function assessments, feelings of “not being well enough at the moment” for additional tests, apprehension related to the risk of contracting the coronavirus (since the study was conducted during the COVID pandemic), and work obligations that limited the duration of patient visits at our hospital. The study was approved by the Ethical Board of Hannover Medical School (no. 6269) and all patients gave written informed consent to participate.

### Assessment of motor function

Motor function of upper extremities and performance in activities of daily living were assessed by the RULM. It is a disease-specific scale, containing 20 items, where the patients can score a maximum of 37 points (higher scores represent better function of upper limbs) [17]. Clinically meaningful changes in RULM were considered if the change in RULM score from baseline to the examined follow-up time point was ≥ 2 [18]. The Hammersmith Functional Motor Scale Expanded (HFMSE) was used to assess patients’ gross motor function. On this 33-item disease-specific scale patients can score a maximum of 66 points, where, again, higher scores represent better motor function [19]. Clinically meaningful changes in HFMSE were defined as a change in HFMSE scores of ≥ 3 [18]. The participants were assessed by trained professional physiotherapists.

### Assessment of treatment satisfaction

To measure patients’ satisfaction with treatment we used the Treatment Satisfaction Questionnaire for Medication German version 1.4 (TSQM) [20]. This 14-item questionnaire is not disease-specific. It can be categorized into four key domains of treatment satisfaction: “effectiveness”, “side effects”, “convenience” and “global satisfaction”. Patients can answer the questions on a five- or seven-point scale (1 meaning extremely dissatisfied, 7 meaning extremely satisfied), except for the dichotomous question 4 (yes or no question). The results for each domain are transformed into scores from 0 to 100, whereby higher scores represent a higher treatment satisfaction. The scores for each dimension were calculated according to the user manual [20].

### Statistical analysis

Statistical analysis was performed using IBM® Statistical Software Package of Social Science (SPSS®, Chicago, IL, USA) version 28. Due to the variable number of cases per time-point and the limited sample size, we abstained from employing descriptive statistical terms such as mean or median, as well as from undertaking subgroup analyses and analyzing differences between baseline and follow-up time points. Correlation between treatment satisfaction and the presence of adverse events were determined with Spearman’s rank (correlation) coefficient.

## Results

### Patients’ characteristics

Table 1 shows the main sociodemographic and clinical characteristics of the enrolled SMA patients at baseline. Eight out of 14 patients were male. Eight patients had SMA type 2, six patients had SMA type 3. Only one patient was ambulatory, most of the patients had scoliosis. Five patients were dependent on NIV, while no patient had a PEG. Prior to initiation of disease modifying treatment, all patients had experienced a subjective steady decline in motor function since symptom onset. Five patients had been on nusinersen treatment prior to risdiplam treatment. Reasons for the switch from nusinersen to risdiplam were severe scoliosis with the necessity of CT-guided lumbar puncture (*n* = 4) and in one patient pronounced discomfort because of the lumbar puncture.

**Table 1:** Clinical and sociodemographic characteristic of SMA patients at baseline

Patient	1	2	3	4	5	6	7	8	9	10	11	12	13	14
Gender	m	f	m	m	f	m	m	f	f	f	f	m	m	m
Age at therapy start (years)	36	51	34	35	34	27	47	27	47	20	39	18	21	23
Previous nusinersen therapy^j^	yes	yes	yes	yes	yes	no	no	no	no	no	no	no	no	no
SMA type	3	2	3	2	2	2	3	3	3	2	3	2	2	2
*SMN2* copy number	4	3	6	3	3	NA	5	NA	NA	NA	NA	3	NA	NA
Ambulatory	no	no	yes	no	no	no	no	no	no	no	no	no	no	no
Scoliosis	no	yes	no	yes	yes	yes	yes	yes	yes	yes	yes	yes	yes	yes
NIV	no	no	no	yes	no	yes	no	no	yes	no	no	yes	yes	no
PEG	no	no	no	no	no	no	no	no	no	no	no	no	no	no
HFMSE score at baseline	8	0	62	1	2	3	3	4	1	NA	6	NA	2	NA
HFMSE score on day of the last nusinersen treatment	9	0	63	1	3	/	/	/	/	/	/	/	/	/
RULM score at baseline	25	13	37	15	9	14	10	19	14	NA	21	NA	1	NA
RULM score on day of the last nusinersen treatment	24	13	37	14	9	/	/	/	/	/	/	/	/	/
Adverse Events during risdiplam therapy	yes^a^	no	yes^b^	yes^c^	yes^d^	yes^e^	yes^f^	no	yes^g^	no	yes^h^	no	yes^i^	

*SMA* spinal muscular atrophy, *m* male, *f* female, *SMN2* *survival of motor neuron 2* gene, *NIV* non-invasive ventilation, *PEG* percutaneous endoscopic gastrostomy, *HFMSE* Hammersmith Functional Motor Scale Expanded, *RULM* Revised Upper Limb Module

*NA* not available, / not applicable

^a^abdominal pain, otitis media

^b^aphthous ulcer, diarrhea

^c^gingivitis, skin rash/increased skin sensitivity to sunlight

^d^constipation, cystitis

^e^diarrhea

^f^diarrhea; g – SARS-CoV-2 infection, abdominal pain,, skin rash/increased skin sensitivity to sunlight

^g^aphthous ulcer

^h^aphthous ulcer

^i^skin rash/increased skin sensitivity to sunlight

^j^patient 1 had 12 doses, patient 2 had 11 doses, patient 3 had nine doses, patient 4 had eight doses and patient 5 had two doses of nusinersen

### Motor function during risdiplam treatment

**Fig. 1:**
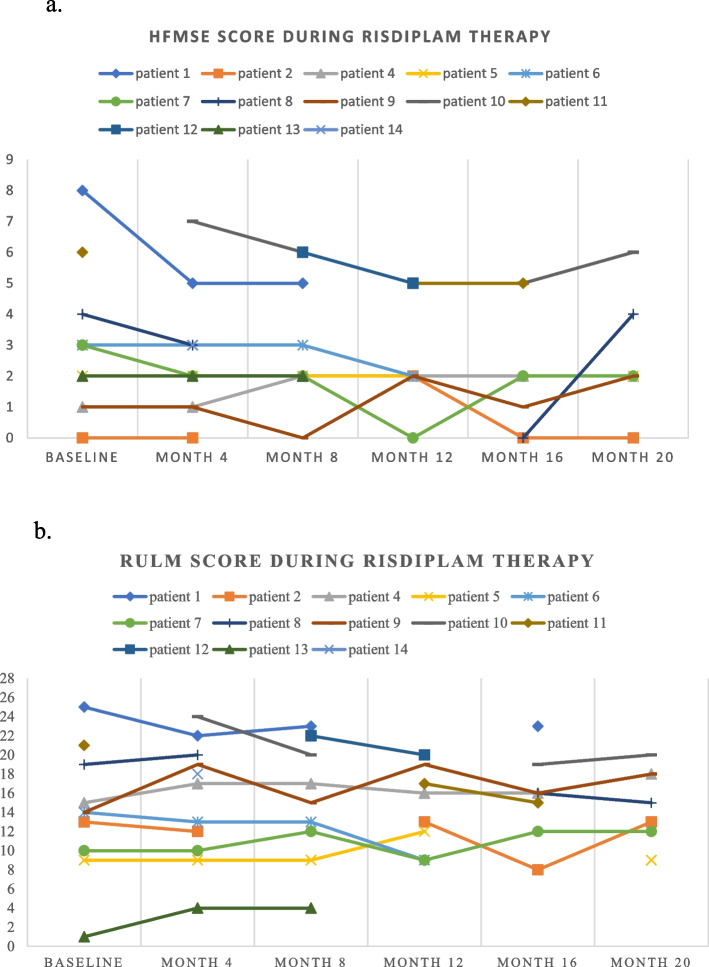
**a** HFMSE score in individual patients during risdiplam treatment. HFMSE – Hammersmith Functional Motor Scale – Expanded; For visibility purposes, the data for patient 3 has been excluded; this patient maintained an HFMSE score of 62 at both baseline and month 4. **b** RULM score in individual patients during risdiplam treatment. RULM – Revised Upper Limb Module; For visibility purposes, the data for patient 3 has been excluded; this patient maintained an RULM score of 37 at both baseline and month 4

Figure 1a and b show the HFMSE and RULM scores of SMA patients throughout the treatment period. Out of 14 patients, half of the patients reached the 20-month follow-up. However, data were incomplete in some patients*.*

Based on the HFMSE score, no patient had clinically meaningful improvement, 12 remained stable, and one experienced clinically meaningful deterioration (patient 1; Fig. 1a). Patient 1 (who showed deterioration) had previously been treated with nusinersen. This patient showed continuous and steady decline in motor function despite disease modifying treatment (HFMSE score = 12 at the start of nusinersen therapy, HFMSE score = 9 at the day of the last nusinersen treatment, HFMSE score = 2 at the day of last risdiplam treatment) (with some fluctuations). He had SMA type 3, four *SMN2* copies, no scoliosis and was non-ambulatory. Additionally, patients 7 and 8 showed transient clinically meaningful deterioration (patient 7 at month 12 and patient 8 at month 16). Both of them had SMA type 3, were non-ambulatory and had scoliosis.

Based on the RULM scores (Fig. 1b), four patients (4, 7, 9, and 13) demonstrated clinically meaningful improvement, three remained stable (patient 2, 3 and 5), and six showed clinically meaningful deterioration (1, 6, 8, 10, 11, and 12). Patient 2 exhibited a transient clinically meaningful deterioration at month 16, while patient 5 showed a transient clinically meaningful improvement at month 12 (Fig. 1b). Patient 7 initially showed clinically meaningful deterioration at month 12 but later clinically meaningful improvement at month 20 (Fig. 1b). Among the patients who improved according to the RULM score, two had SMA type 2, two had SMA type 3, none were ambulant, and all had scoliosis. Among those who showed clinically meaningful deterioration, three had SMA type 2, three had SMA type 3, none were ambulant, and only one had no scoliosis.

### Adverse events during risdiplam treatment

There was no treatment withdrawal during the follow-up period and all participants intended to further continue treatment with risdiplam at the end of the study. Short-lasting adverse events were present in nine patients at some time during the treatment period (Table 1). The most frequently reported adverse events were skin rash/increased skin sensitivity to sunlight (*n* = 3), diarrhea (*n* = 3), aphthous ulcer (*n* = 3) and abdominal pain (*n* = 2). Constipation, otitis media, cystitis and gingivitis were present only as single short-lasting events during risdiplam treatment. None of the patients had to be hospitalized. Five patients had mildly elevated liver transaminases (less than 1.5 × the upper limit of normal) which subsequently normalized without any specific treatment.

### Treatment satisfaction during risdiplam treatment

**Fig. 2:**
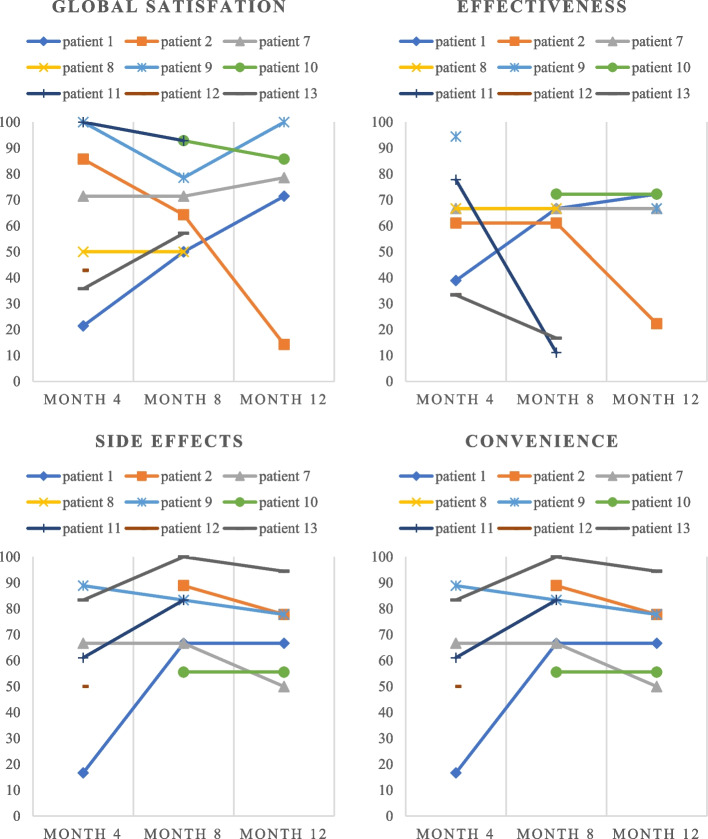
Domains of treatment satisfaction during risdiplam treatment

Figure 2 shows the change in “global satisfaction”, “side effects”, “effectiveness” and “convenience” during risdiplam treatment. Seven patients reported to be at least “somewhat satisfied” with the medication (four were very satisfied or extremely satisfied) at month 4. All patients reported to be at least “somewhat satisfied” with the medication (three were very satisfied) at month 8. Only one patient reported to be very dissatisfied with the treatment, while the rest stated to be at least “satisfied” with the medication at month 12. Despite this lack of satisfaction, this patient remained clinically stable (no clinically meaningful change in HFMSE or RULM score) at month 12.

We observed no correlation between treatment satisfaction and the presence of adverse events during the treatment or the change in RULM/HFMSE score during risdiplam therapy (*p* > 0.05).

## Discussion

This is one of the first studies in a real-world setting in adult SMA patients that describes motor function under treatment with risdiplam. Furthermore, the present study is the first one also including SMA type 3 patients, so far, with the biggest cohort and longest follow-up period. Our data indicate that risdiplam is in general well tolerated and somewhat effective.

According to the HFMSE score, almost all patients (*n* = 12) remained stable and only one exhibited a permanent deterioration during the follow-up of up to 20 months. Additionally, two patients showed a transient deterioration throughout the treatment period. Most of our patients demonstrated a notably low baseline motor function based on the HFMSE. Given the progressive decline of motor function in the natural history of SMA [21, 22], and the fact that no improvements but rather stabilization were found in the SUNFISH trial in the oldest age group (18–25 years) [13], improvements in the HFMSE cannot be anticipated. The RULM score demonstrated greater responsiveness in detecting changes in the motor function of the upper extremities in our SMA patients. About half of the patients (*n* = 7) remained stable or even improved, while six patients experienced a deterioration. Stabilization as well as improvement on both HFMSE and RULM should be regarded as treatment success given that natural history studies indicate significant declines of motor function over time, with a mean change of -1.71 for HFMSE at month 36 [22] and a mean change of -0.41 for RULM at month 12 [23].

Contrary to our results, Ñungo Garzón et al. reported no clinically meaningful deteriorations in RULM (worsening of RULM score ≥ 2 vs. baseline) during their 12 months follow-up period of six SMA type 2 patients [15]. A possible explanation for this might be a floor effect in their study as half of the patients scored 0 for their baseline RULM assessment [15]. McCluskey et al. reported clinically meaningful improvement in RULM scores in two patients and stabilization in the other three (*n* = 6, one patient lost to follow-up) [14]. Our results are in line with the SUNFISH trial, where no improvement was seen 12 months after treatment initiation in the oldest examined age group (18–25 years) [13]. In the latest report from the SUNFISH trial, 52% of patients of the whole cohort (aged 2–25 years) exhibited an improvement in RULM score after 24 months of risdiplam treatment [24]. Four out of five patients previously treated with nusinersen in our cohort remained stable (according to the HFMSE score) after the switch to risdiplam, while one patient (patient 1; Fig. 1a) experienced a clinically meaningful deterioration. In the study of Ñungo Garzón et al., two patients had previously been treated with nusinersen. Out of them, one patient remained stable, while the other one showed clinically meaningful improvement (improvement of RULM score ≥ 2 vs. baseline) after 12 months of treatment [15]. The assessment of respiratory and bulbar function was not conducted in our study, and it would be recommended that future studies place emphasis on these aspects as well. If risdiplam proves effective in halting the deterioration of bulbar or respiratory function in adult SMA patients, this would represent a meaningful treatment benefit, even in the absence of notable improvements of gross motor function.

Risdiplam was well-tolerated in our study, with adverse events reported by about two-thirds of patients. The most common events, reported by three patients each, were skin rash/increased skin sensitivity to sunlight, diarrhea, and aphthous ulcer. Contrary to this, in the study of McCluskey et al., all the included patients (*n* = 6) had skin rash [14], while other side effects were nephrolithiasis (< 20%), diarrhea/constipation (< 20%) and elevated liver transaminases (< 20%). In our study, almost one third of the patients showed mildly elevated liver transaminases (less than 1.5 × the upper limit of normal) which subsequently normalized without any specific treatment. Two recent real-world studies (one from Germany that included 36 SMA type 1 and 98 SMA type 2 patients, and one from the USA that included 73 SMA type 1 and 82 SMA type 2 patients) on safety of risdiplam in children and adults with SMA reported that the most common treatment related adverse events were diarrhea, nausea, constipation, rash, and headache [25, 26].

Throughout one year of risdiplam treatment, patients’ “global satisfaction” remained relatively stable and most of the patients reported to be at least “somewhat satisfied” with the medication. Patients reported the highest satisfaction with “side effects”, even though side effects were reported by 64% of included patients. Interestingly, patients’ satisfaction with “convenience” in our cohort was higher (all patients scored ≥ 50.0 at month 12; Fig. 2) in comparison to a previous study in 91 mainly adult SMA patients treated with nusinersen, where a mean of 43.6 ± 20.2 at month 10 of nusinersen treatment was observed [27]. The need for repeated lumbar punctures and frequently CT-guided nusinersen administration might be the most plausible explanation. In our study we observed no correlation between treatment satisfaction and the presence of adverse events during the treatment or the change in RULM/HFMSE score during risdiplam therapy. This could be due to small sample size and loss of follow-up data. Therefore, future studies in larger cohorts of SMA patients should correlate the domains of patients’ satisfaction with motor function, adverse events and quality of life.

The relatively small number of patients due to the monocentric design of the study, absence of a control group and loss of follow-up data are the main limitations of this study. Additionally, information about the *SMN2* gene copy number was unavailable for some patients. However, this study had the longest follow-up period in a real-world setting so far and, for the first time, included patients with SMA type 3, one of them being ambulatory. Studies including larger numbers of patients with a prospective design are needed to fully assess the impact of risdiplam on motor function as well as treatment satisfaction in adult SMA patients.

In conclusion, risdiplam was well tolerated and motor function remained stable or improved in half of the patients after risdiplam initiation. Patients were generally satisfied with the treatment throughout the analyzed treatment period. Larger and multicentric studies are needed in order to draw more relevant conclusions.

## Data Availability

The datasets used and/or analysed during the current study are available from the corresponding author on reasonable request.

## Abbreviations

HFMSE: Hammersmith Functional Motor Scale Expanded
NIV: Non-invasive ventilation
PEG: Presence of percutaneous endoscopic gastrostomy
RULM: Revised Upper Limb Module Hammersmith Functional Motor Scale Expanded
SMA: Spinal muscular atrophy
SMN: Survival of motor neuron
*SMN2*: *Survival of motor neuron 2 gene*
TSQM: Treatment Satisfaction Questionnaire for Medication
